# Supplementary figures and images for: Analysis of the release pattern of floral aroma components of Rhus chinensis based on HS-SPME-GC-MS technique
Source: PLoS One. 2025 Mar 12;20(3):e0319211. doi: 10.1371/journal.pone.0319211 (PMC11902258; doi:10.1371/journal.pone.0319211)

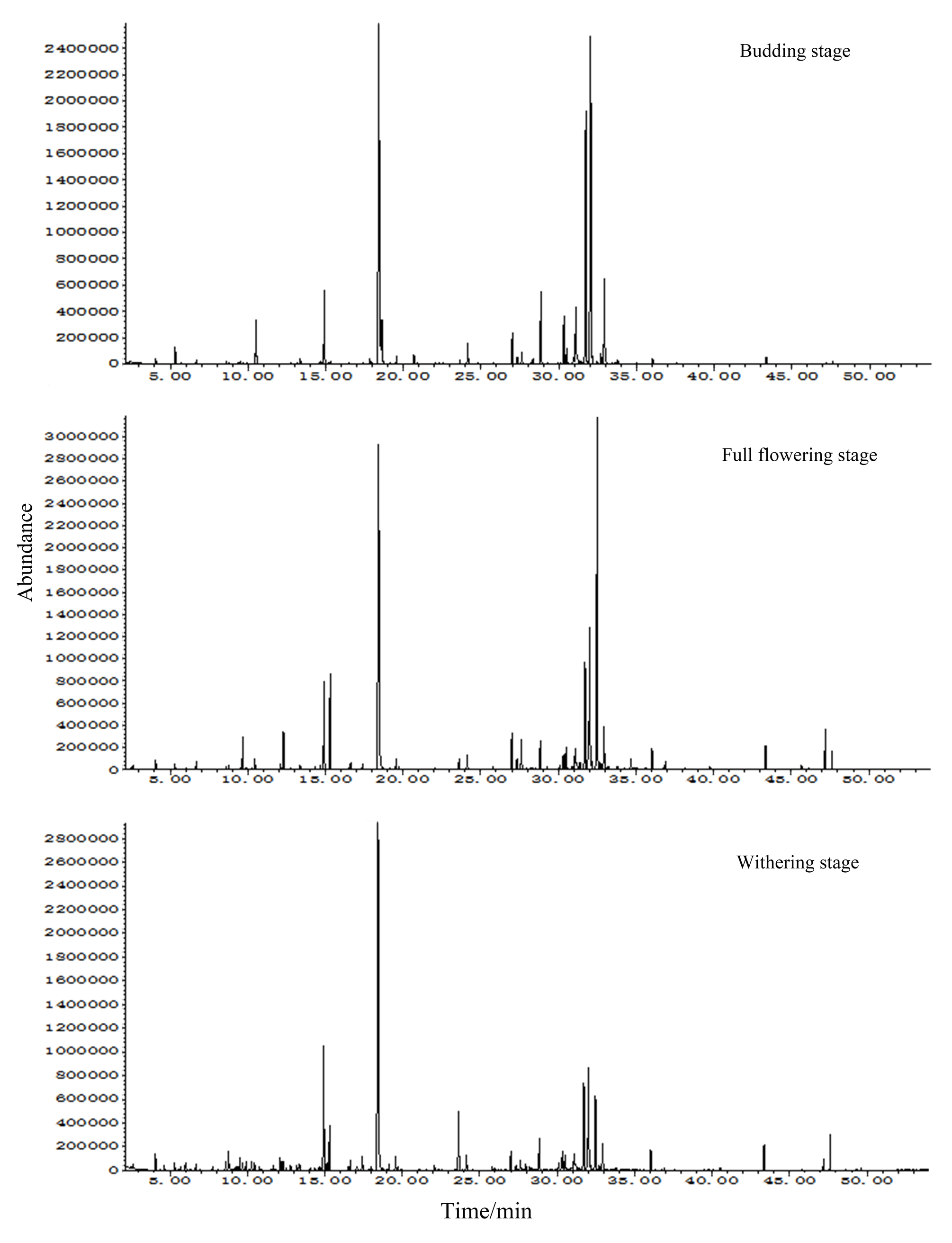

Supplement: S1 Fig — (TIF) [file pone.0319211.s001.tif]

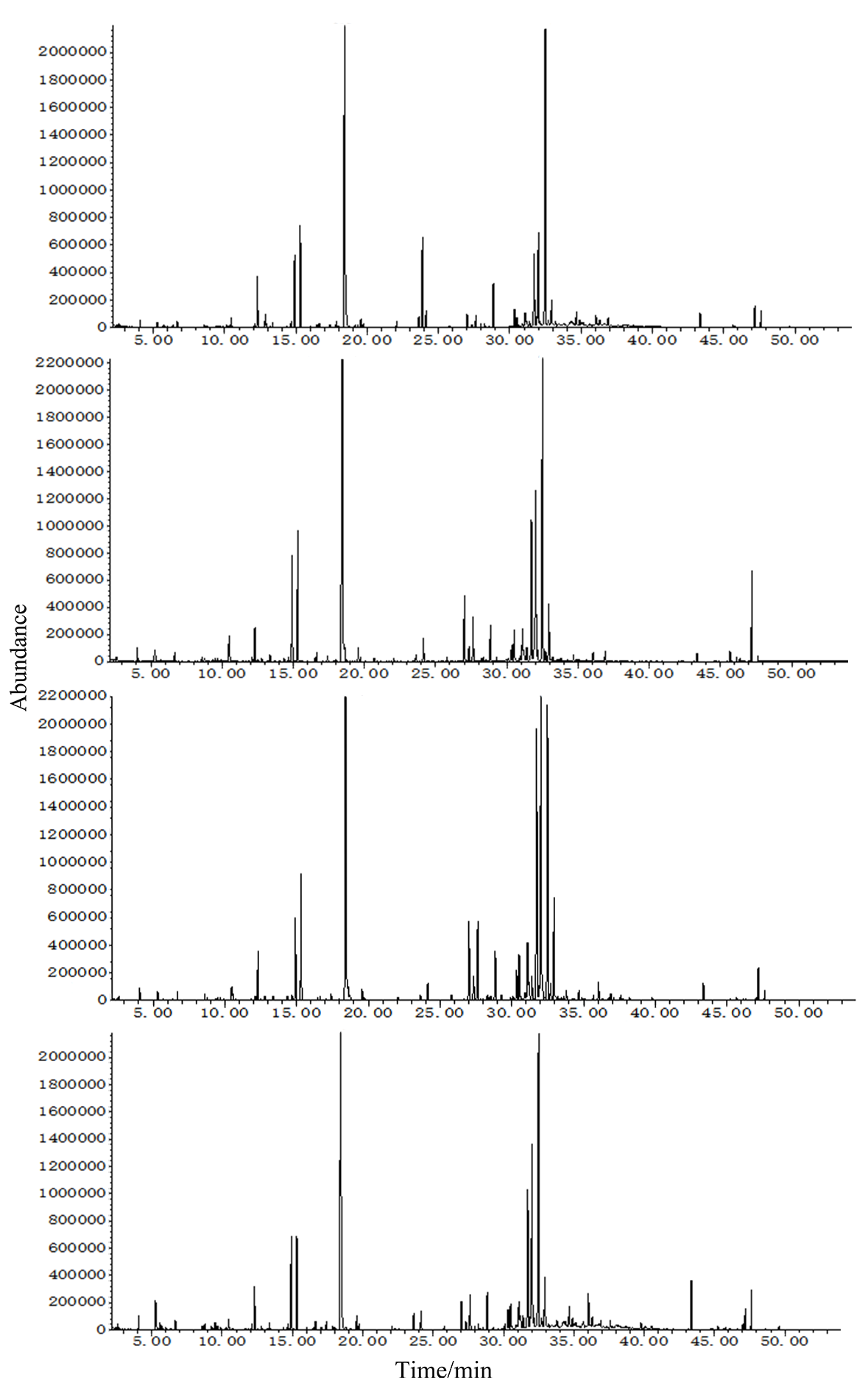

Supplement: S2 Fig — (TIF) [file pone.0319211.s002.tif]
